# Early life stress, lower urinary tract dysfunction, and respiratory disease: a proposed shared-mechanism framework

**DOI:** 10.3389/fphys.2026.1929695

**Published:** 2026-10-12

**Authors:** Tadanobu Chuyo Kamijo, Mayoko Tsuji

**Affiliations:** 1 Department of Systems Physiology, Graduate School of Medicine, University of the Ryukyus, Ginowan, Okinawa, Japan; 2 Department of Respiratory Medicine, Tokyo Women’s Medical University, Tokyo, Japan

**Keywords:** asthma, early life stress, epigenetics, extracorporeal shock wave therapy, gut–lung–bladder axis, HPA axis, lower urinary tract symptoms, maternal separation

## Abstract

Early life stress (ELS) has been associated with persistent dysfunction across multiple physiological systems in humans, and maternal separation produces such dysfunction in rodent models. The respiratory and urinary tracts appear functionally distinct, yet both may be influenced by candidate overlapping neuro-endocrine, immune, and epigenetic consequences of ELS — a convergence that has received little attention because respiratory medicine, urology, and stress physiology have largely studied these effects in isolation. Our starting point is an experimental observation: in maternal separation rats, bladder-directed low-intensity extracorporeal shock wave therapy (Li-ESWT) reduced the elevated bladder weight and also improved anxiety-like behavior, showing that an intervention directed toward the bladder — regional rather than strictly organ-restricted — can be followed by change in a central behavioral endpoint, although the route, direction, and causality were not established. If peripheral-to-central communication of this kind is general, organs other than the bladder that may share elements of ELS-related programming could be similarly accessible. The airway is a plausible candidate: in mouse studies of maternal separation, ELS suppresses regulatory T-cell development and biases airway immunity toward Th2 responses, while separate rat studies report bladder overactivity, altered voiding, and anxiety-like behavior. The two have not been measured in the same animals. We review the candidate shared substrates — hypothalamic–pituitary–adrenal (HPA) axis dysregulation, chronic low-grade inflammation (e.g. IL-6, TNF), autonomic/vagal imbalance, microbiome-mediated immune programming along the gut–lung and gut–bladder axes, and epigenetic reprogramming — distinguishing throughout what has been shown in humans from what rests on rodent models. We propose a “psycho-neuro-immune-urinary-respiratory nexus” as a testable framework rather than an established entity, and outline the experimental models, biomarkers, and integrated therapeutic strategies needed to test it.

## Introduction

### Early life stress as a multi-organ phenomenon

Early life stress (ELS) extends beyond any single organ system, producing profound and lasting effects across the body. ELS has been associated with alterations in central regulatory systems — the HPA axis (Nicolaides et al., 2024), the autonomic nervous system (Gapen and Guy, 2021), and the immune system (Jessop, 2008) — and has been reported, in separate literatures, to affect organ systems as seemingly unrelated as the respiratory and urinary tracts. Because each clinical field has examined these effects within its own boundaries, a possible common pathophysiological foundation has remained underappreciated.

In rodent models, ELS such as maternal separation induces structural and functional changes in the developing brain (Guadagno et al., 2021) and cognitive–emotional abnormalities that persist into adulthood (González-Acosta et al., 2021). In humans, early adversity is followed by lasting recalibration of HPA-axis reactivity (Gunnar et al., 2019). HPA-axis dysregulation, together with epigenetic change, is the route most commonly proposed for long-term multi-organ consequences, although that mediation has not been demonstrated directly. Importantly, ELS shifts inflammatory cytokine production (Tian et al., 2014), elevating IL-6 and TNF (Jessop, 2008); this chronic inflammatory tone has been associated with airway hypersensitivity and asthma risk in the lung (Chida et al., 2007) and with bladder dysfunction and pain in the urinary tract (Chess-Williams et al., 2021). ELS has also been associated with reduced heart rate variability and altered autonomic balance (Kim et al., 2022). This developmental trajectory — a critical early window, followed by functional change in both the airway and the bladder that persists into adulthood — is summarized in **Figure 1**.

**Figure 1:**
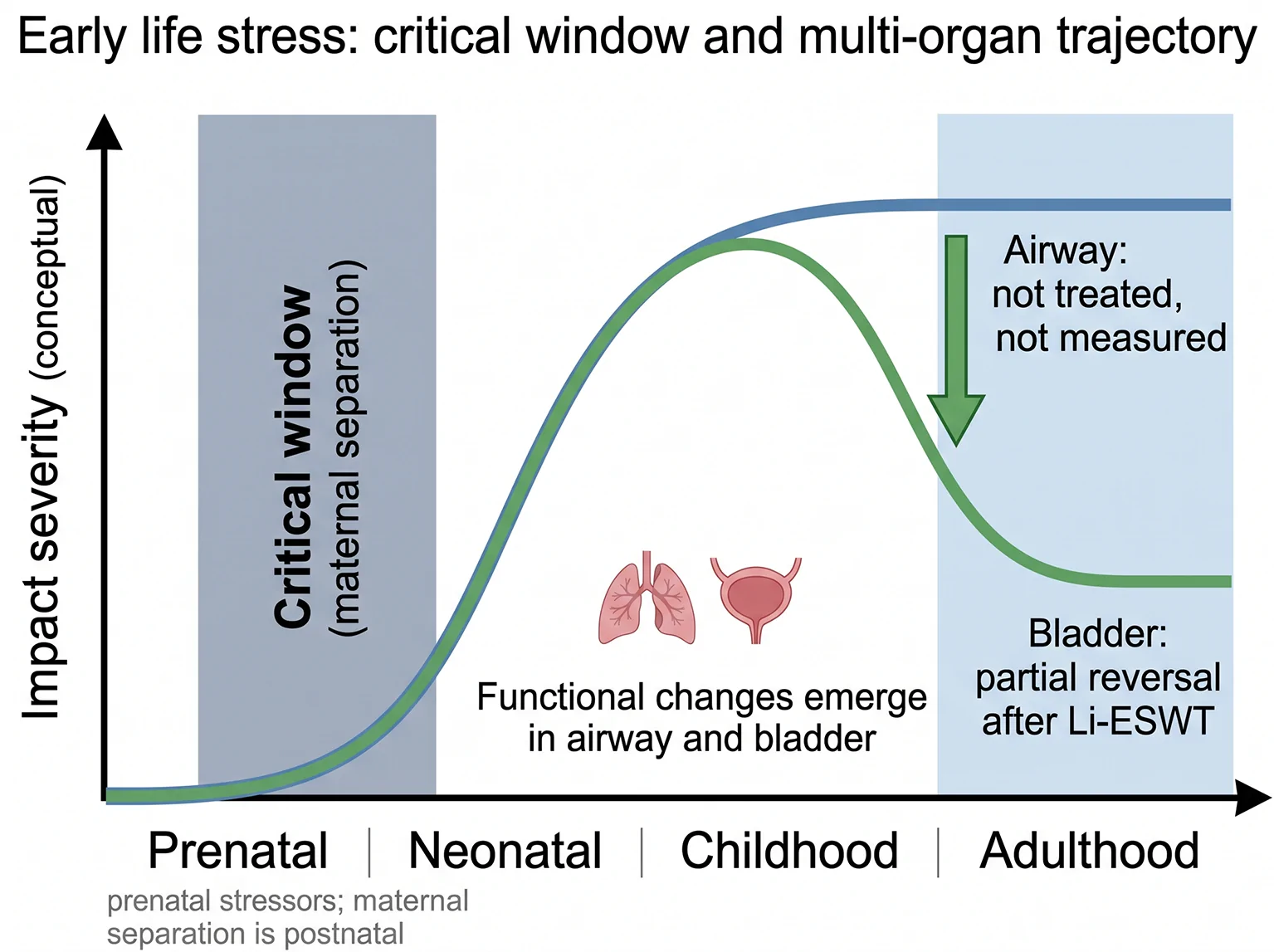
Early life stress: critical window and multi-organ trajectory. The schematic summarizes the account developed in this review: an early developmental window during which stress exposure has disproportionate effect, followed by functional change in the airway and in the bladder that persists into adulthood. The two traces run together through early development and separate toward adulthood: the upper trace is the airway, which was neither treated nor measured, and the lower trace is the bladder after treatment. Evidence for the airway and evidence for the bladder come from separate rodent studies; the two phenotypes have not been measured in the same animals. The partial reversal indicated at the adult stage refers to bladder outcomes only, following bladder-directed low-intensity extracorporeal shock wave therapy (Li-ESWT); no respiratory Li-ESWT data exist in this context. The prenatal segment refers to the wider early-life-stress literature, which includes prenatal stressors, rather than to maternal separation, which is a postnatal manipulation. The figure is conceptual: the vertical axis is not a measured quantity, and quantitative values are given in the text.

### Convergent evidence in both urinary and respiratory disease

Animal models show that ELS affects both systems. Maternal separation alters voiding behavior and bladder activity from early maturation, with bladder overactivity and anxiety-like behavior co-occurring into adulthood (Kamijo and Miyazato, 2023); it likewise impairs respiratory tolerance, suppressing regulatory T-cell generation and enhancing allergic airway responses in adulthood (Ouchi et al., 2018).

Clinical data echo this convergence. Adverse childhood experiences (ACEs) are associated with a higher probability of developing multiple asthma phenotypes in a national cohort (Pape et al., 2021), and prenatal/early-life stress is repeatedly linked to later asthma and other respiratory disease, potentially involving immune and neuroendocrine mechanisms (Rosa et al., 2018; Lee et al., 2016). On the urinary side, ACEs and childhood trauma are associated with adult lower urinary tract symptoms (LUTS). In the CARDIA cohort, each unit increase in ACE frequency raised the odds of any LUTS/impact by 34% (OR 1.34, 95% CI 1.11–1.62) (Brady et al., 2023). The LURN study was more specific: total childhood trauma was not associated with overall LUTS, but childhood sexual trauma was associated with greater urinary incontinence severity (adjusted mean difference 4.5 points, 95% CI 1.11–7.88, p = 0.009), with roughly half of that effect mediated through mental-health pathways (Geynisman-Tan et al., 2021). Consistently, 30% of overactive-bladder patients report childhood sexual trauma, and childhood trauma predicts worse bladder and non-urologic pain, poorer mood, and greater somatic and psychological-stress burden — though, as in LURN, not the severity of core voiding symptoms (urgency, frequency, incontinence) (Lai et al., 2016). Together these observations suggest shared neurobiological pathways linking stress, inflammation, and organ dysfunction (Gkesoglou et al., 2022).

Epigenetic mechanisms offer a candidate route to multi-organ ELS effects: DNA-methylation changes (Nicolaides et al., 2024), histone modifications, and microRNA changes (McKibben and Dwivedi, 2021) have each been reported to alter stress- and inflammation-related gene expression. Whether all three operate persistently and concurrently across the organ systems considered here has not been shown.

### Why the fields have remained separate

Respiratory medicine, urology, and psychiatry have each characterized ELS effects in depth but with limited cross-talk. Three barriers stand out. The animal models and endpoints are field-specific (Schneider et al., 2017). The standard functional assays in each field were developed independently, so respiratory and urinary function are rarely measured in one experiment (for the respiratory assays, see Polikepahad et al., 2010). And few clinical tools assess multi-organ stress effects integratively (Bali and Jaggi, 2015). Therapeutically, each field is symptom- and organ-specific — asthma care targets airway inflammation (e.g. triple therapy for cough in the COCOA trial; Tagaya et al., 2025), urinary care targets bladder function — and integrative strategies aimed at a putative shared neuro-immuno-endocrine substrate are rare.

### The proposed nexus: definition, status, and boundaries

We use the term “psycho-neuro-immune-urinary-respiratory nexus” to denote a single proposition: that early life stress may install a set of shared upstream regulatory changes — in HPA-axis signaling, autonomic and vagal control, immune set-point, the early-life microbiome, and the epigenome. On this view, the respiratory and lower urinary tract phenotypes observed after ELS may be downstream expressions of that shared upstream state rather than independent organ-level lesions (**Figure 2**). We advance this as a testable hypothesis, not as an established biological entity and not as a clinical framework ready for use. Its novelty lies not in any individual mechanism, each of which is already documented within its own field, but in what follows if they are treated as one substrate.

**Figure 2:**
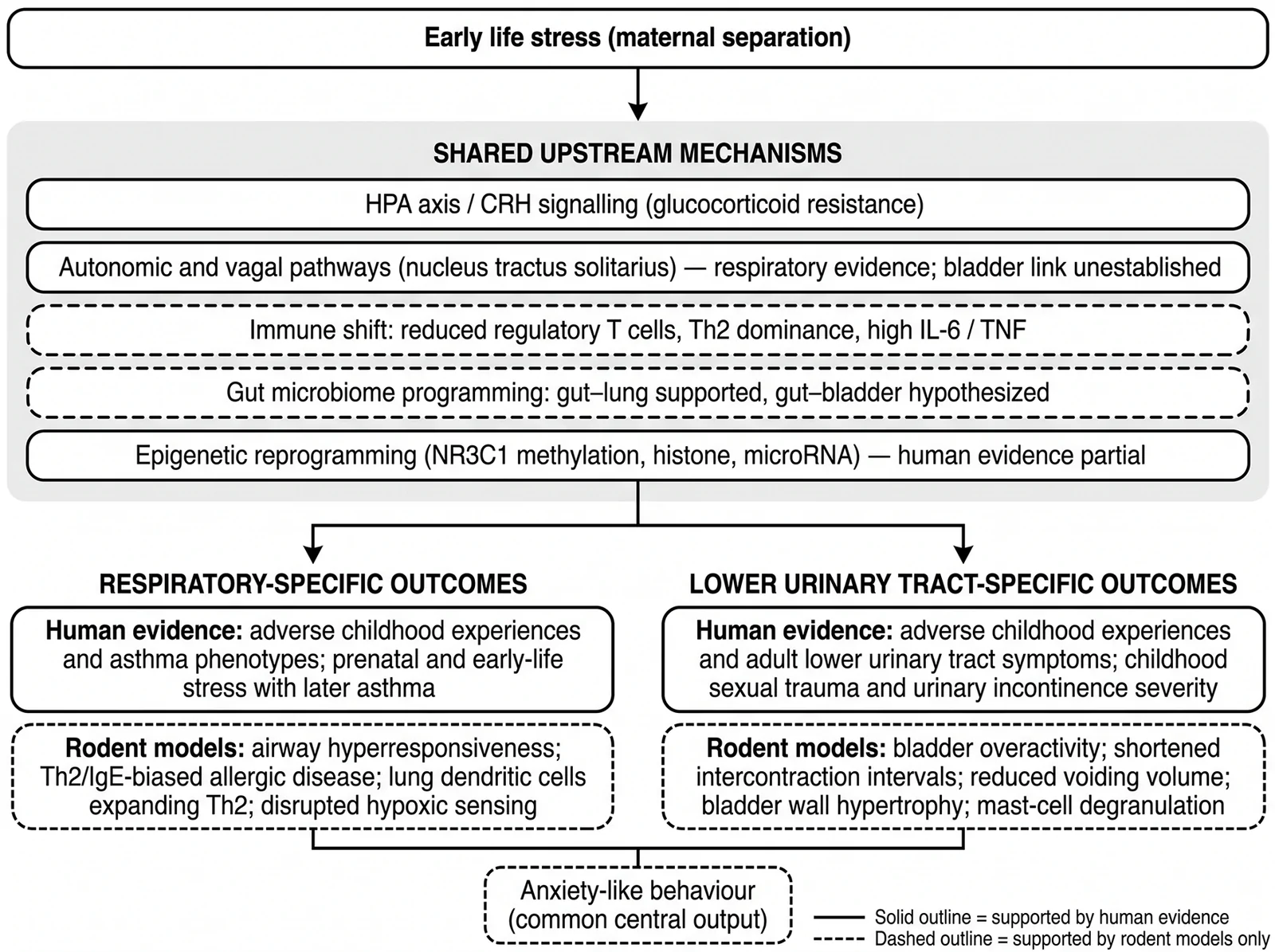
Shared upstream mechanisms versus organ-specific outcomes after early life stress. Maternal separation is proposed to act on a set of candidate shared upstream mechanisms — HPA-axis/CRH signaling with glucocorticoid resistance, autonomic and vagal pathways including the nucleus tractus solitarius, an immune shift toward reduced regulatory T cells with Th2 dominance and elevated IL-6/TNF, gut microbiome programming, and epigenetic reprogramming (NR3C1 methylation, histone modification, microRNA) — which then diverge into respiratory-specific and lower urinary tract-specific outcomes, with anxiety-like behavior as a common central behavioral output. For each organ system the outcomes are shown in two parts, those established in humans and those resting on rodent models. Solid outlines mark elements supported by human evidence; dashed outlines mark elements resting on rodent models only. Individual boxes carry their own qualifications where the evidence is uneven: the gut–lung microbiome link is better supported than the gut–bladder link, the nucleus tractus solitarius evidence comes from the respiratory side with no established vagal link to the bladder, and the human epigenetic evidence is only partial. The figure separates what the framework proposes to be shared from what is organ-specific, and is offered as a hypothesis rather than as an established pathway.

The primary prediction concerns concordance. A usable test requires one upstream index to be nominated in advance rather than chosen afterwards. We would nominate the regulatory T-cell fraction, because it is the candidate for which an ELS effect has been demonstrated on the respiratory side, and because it can be measured in the same animal as the two organ phenotypes. The test also requires defined airway and voiding endpoints, the two sexes analyzed separately, a matched developmental stage, and a non-ELS comparison group. Under those conditions the two phenotypes should co-vary with one another and with the index, and restoring the index should move both, whereas perturbing a pathway specific to one organ should move only that organ. The proposition is weakened if the phenotypes fail to co-vary within sex and stage in a design powered to detect it, and it is contradicted if restoring the index reliably moves one organ and not the other. Two failure modes have to be allowed for. A genuinely shared upstream cause can still produce discordant organ effects through differences in threshold, timing, or local effect modifiers, so discordance alone is weaker evidence against the proposition than concordance is for it. To keep that caveat from becoming an unfalsifiable escape, we state in advance what would settle it: if restoring the index moves one organ phenotype and leaves the other unchanged in both sexes, at more than one dose, and at more than one developmental timepoint, we would treat the proposition as contradicted rather than merely qualified. Conversely, a manipulation that moves both organs may act through a nonspecific systemic route rather than through ELS-related programming; the non-ELS comparison group is what separates these, since there the same manipulation should not reproduce the paired phenotype.

A separate question, which is not itself a prediction of the nexus, is whether an intervention directed toward one peripheral organ can produce change at a distant site; the observation that comes closest to this — bladder-directed Li-ESWT improving bladder dysfunction and, alongside it, an anxiety-like behavioral endpoint in maternal-separation rats — is what motivated this review. Because such an effect could also arise through routes that do not require shared early-life programming, including activation of local sensory afferents, it does not on its own test the proposition above.

We also state what the hypothesis does not claim. We do not claim that causality has been demonstrated in humans; that respiratory and urinary phenotypes have been measured in the same subjects; that a gut–lung–bladder axis has been shown to operate as an integrated pathway; or that Li-ESWT has been shown to treat ELS-related disease beyond the bladder. Where evidence is confined to rodent models, we say so; **Figure 2** applies the same distinction graphically, using solid outlines for elements supported by human evidence and dashed outlines for those resting on rodent models alone.

### Aim

This review integrates current knowledge on the interconnected respiratory and urinary effects of ELS and examines the candidate common mechanisms that might underlie multi-organ dysfunction. We (1) examine the interacting neural, immune, microbiome, and epigenetic mechanisms; (2) examine candidate experimental interventions — principally Li-ESWT — that could be used to probe cross-organ effects; and (3) propose research directions that would test the framework. Spanning respiratory medicine, urology, and stress physiology, we aim to clarify how early adversity may create durable cross-organ vulnerability, and to set out what would have to be shown for that account to hold.

## Maternal separation and the urinary system

### Neural development and lower urinary tract function

In rodent models, neonatal maternal separation alters development of the neural circuits controlling voiding, and is followed by lower urinary tract dysfunction that persists into adulthood. Normal voiding requires coordinated maturation of brainstem voiding centers, sacral reflexes, and cortical control, and this circuitry remains plastic through development and after injury (Hsiang and Vizzard, 2024). That plasticity is why an early insult is a plausible route to lasting dysfunction, but whether maternal separation acts on these maturational processes directly has not been demonstrated. Altered PACAP/PAC1 neuropeptide signaling accompanies increased voiding frequency and urgency (Gonzalez et al., 2016; Ojala et al., 2018).

### Findings from maternal separation models

In a study of 85 rat pups, maternal separation reduced single voiding volume (both sexes, 3 weeks) and shortened intercontraction intervals (both sexes, 9 weeks), with bladder weight/wall changes in 3-week females and bladder wall hypertrophy in 9-week males (Kamijo and Miyazato, 2023). Neonatal maternal separation also increases bladder sensitivity, voiding frequency, and mast-cell degranulation; voluntary wheel running normalizes bladder sensitivity and voiding volume, indicating partial reversibility (Pierce et al., 2018).

Behavioral changes track the urinary phenotype. In the same cohort, elevated plus-maze testing showed increased anxiety-like behavior at 3 weeks (reduced open-arm entries and speed) that shifted by 9 weeks, demonstrating developmental-stage-dependent responses (Kamijo and Miyazato, 2023). Early stress more broadly produces neurogenic inflammation and bladder hypersensitivity, with increased voiding frequency and reduced capacity (Fuentes et al., 2018; Mingin et al., 2014).

### Sex differences

Urinary effects of ELS are sexually dimorphic, with females generally more affected in animal models. Female rats exposed to unpredictable ELS showed reduced detrusor contractility, whereas males showed little change (Mohammadi et al., 2016). The dimorphism is not uniform across endpoints, however: in male mice, neonatal maternal separation produced urogenital neurogenic inflammation and hypersensitivity that was exacerbated by later adult stress (Fuentes et al., 2018), so that mechanism is not female-specific. α1A-adrenoceptor and TRPV1 involvement has also been described in the urinary tract (Matos et al., 2024). Sex- and hemisphere-dependent hippocampal neurochemistry may contribute (McQuillan et al., 2024). Sex hormones themselves shape lower urinary tract structure and function: in a mouse model of menopause-induced lower urinary tract dysfunction, declining estrogen altered voiding characteristics, bladder wall structure, and related protein expression (Zhang C. et al., 2024). This indicates that estrogen status alone can shape these endpoints; whether it accounts for the female predominance seen after early stress has not been tested.

### Reversibility through peripheral intervention

Maternal-separation–induced bladder and behavioral changes can be ameliorated by peripheral therapy. Bladder-directed Li-ESWT (0.12 mJ/mm², 2 Hz, 200 shocks/session, nine sessions, from 6 weeks of age) reduced bladder weight in maternal-separation (hereafter MS) rats to values not significantly different from control. In males, bladder weight was 151.6 ± 7.6 mg in normal animals, 211.5 ± 17.4 mg in MS, and 169.6 ± 7.3 mg after treatment; in females the corresponding values were 113.0 ± 7.8, 167.0 ± 6.2, and 100.3 ± 2.6 mg (means ± SEM, n = 8 per sex per group). In the mixed-sex analysis the group effect was significant (F(2,45) = 11.19, p = 0.0001; normal vs MS p < 0.001; MS vs MS+Li-ESWT p < 0.001; normal vs MS+Li-ESWT not significant). Bladder weight is used here as an index of wall remodeling: maternal separation raised it by approximately 40% in males and 48% in females, and after treatment it no longer differed significantly from control in either sex. Body weight showed no significant group difference in the mixed-sex analysis, so no difference in body size was detected that could account for the bladder-weight effect. A non-significant comparison does not exclude body size as a contributor, and the study did not normalize bladder weight to body weight. Two controls strengthen the interpretation: Li-ESWT applied to normal animals did not significantly alter bladder weight, and no group difference was seen in adrenal weight in the mixed-sex analysis.

Treatment also reduced anxiety-like behavior, with restored open-arm time on the elevated plus maze (Kamijo et al., 2025). Behavioral improvement was more pronounced in females, with greater recovery of anxiety-like behavior and of dark-phase voiding frequency, whereas the bladder-weight increase was larger in absolute terms in males (+60 mg) and larger in relative terms in females (+48% versus +40%); the changes occurred without gross histological change, and the mediating mechanism was not measured. Indirect support comes from a different disease model: in capsaicin-induced prostatitis rats, low-intensity shock wave therapy was associated with neurotrophic factor modulation (BDNF/NGF downregulation in dorsal root ganglion and spinal cord) and with reduced BOLD responses in pain-related regions (Wang et al., 2022). Whether the same processes operate after bladder-directed treatment in ELS animals has not been tested. A plausible substrate is Barrington’s nucleus, where social stress upregulates CRF and a CRF1-receptor antagonist reverses the resulting urodynamic dysfunction (Wood et al., 2013; discussed further below); whether it participates in any peripheral-to-central effect of Li-ESWT has not been examined.

## Maternal separation and respiratory disease

### Early-life stress and asthma risk (epidemiology)

ACEs are associated with the development of multiple asthma phenotypes in large cohorts (Pape et al., 2021), and both prenatal and early-childhood stress correlate with later asthma, potentially involving immune and neuroendocrine dysfunction (Rosa et al., 2018). The developing fetus is especially susceptible during rapid lung morphogenesis, when stress can alter immune and antioxidant defenses (Lee et al., 2016).

### Asthma susceptibility in mouse models

Maternal separation impairs respiratory tolerance and enhances adult allergic airway responses (Ouchi et al., 2018): MS mice show airway hypersensitivity, elevated allergen-specific IgE, and Th2-biased cytokines, attributable to insufficient regulatory T-cell development. Early-life antibiotic exposure similarly disrupts the gut microbiota and durably enhances allergic airway inflammation, even after microbiome normalization (Perdijk et al., 2024) — a direct link to the microbiome axis developed below.

### Treg/Th2 balance and pulmonary inflammation

In a maternal-separation mouse model, ELS suppresses generation of regulatory T cells that maintain tolerance to inhaled allergens (Ouchi et al., 2018). Th2 dominance, with overproduction of IL-4, IL-5, and IL-13 and elevated IgE, is the pattern that stress has been associated with more broadly in the earlier literature (Chida et al., 2007), although that review does not establish this specific cellular sequence. Prenatally stressed offspring likewise show a Th2-skewed cytokine profile and elevated serum IgE, with lung dendritic cells that preferentially expand Th2 cells (Pincus-Knackstedt et al., 2006). Pro-inflammatory cytokines, including IL-6 and TNF, have separately been implicated in bladder sensitivity and function (Chess-Williams et al., 2021; Girard et al., 2023). Whether the elevations reported in the airway studies and those reported in the bladder studies reflect one shared substrate is not established, and the two have not been measured in the same subjects.

### Sex as a modulator

Respiratory effects of maternal separation are sex-dependent: males show increased apnea and altered hypoxic/hypercapnic ventilatory responses, with distinct female patterns (Tenorio-Lopes and Kinkead, 2021; Elliot-Portal et al., 2021). Epidemiologically, boys appear more susceptible to prenatal stress and girls to postnatal stress (Lee et al., 2016); hormonal and immune-developmental differences are the usual explanation offered, but that account has not been tested against this pattern. Cerebral erythropoietin has been proposed to mitigate ELS effects on respiratory control (Elliot-Portal et al., 2021).

## Integrated neuro-immuno-endocrine and microbiome mechanisms

### HPA axis and CRH neurons

The HPA axis coordinates multi-organ stress responses, integrating neuroendocrine, cardiovascular, and immune function (Smith and Vale, 2006). CRF drives glucocorticoid release with widespread effects (Herman et al., 2012). Acutely, glucocorticoids and catecholamines suppress pro-inflammatory cytokines (Tian et al., 2014), but chronic stress produces glucocorticoid resistance and elevated IL-6/TNF, worsening inflammatory disease (Jessop, 2008; Martinez et al., 2024).

CRF couples the axis to both target organs. CRF is a potent respiratory stimulant: intravenous human CRF increased respiratory minute volume by 81% in healthy volunteers (Oppermann et al., 1986), an effect that persists under dexamethasone and thus appears independent of cortisol (Huber et al., 1989). On the urinary side, social stress produced urinary retention and urodynamic abnormality with CRF upregulation in Barrington’s nucleus, reversed by a CRF1-receptor antagonist (Wood et al., 2013). A single preclinical study bears on this: bladder-directed Li-ESWT improved bladder dysfunction and, alongside it, an anxiety-like behavioral endpoint in MS rats (Kamijo et al., 2025); we return to that result, and to its limits, below. Related work in a capsaicin-induced prostatitis model reports downregulation of stress-related neurotrophic factors in dorsal root ganglion and spinal cord together with reduced BOLD responses in pain-related regions (Wang et al., 2022), but that is a different model, and the molecular basis of any peripheral-to-central effect in ELS animals remains unestablished.

### Inflammation–vagus–cytokine crosstalk

ELS engages inflammatory networks via the HPA axis and sympathetic nervous system; elevated cytokines during critical windows impair neural development and behavior (Paraschivescu et al., 2022). The vagus nerve, through the nucleus tractus solitarius, integrates respiratory afferents and regulates autonomic output (Zoccal et al., 2014). Whether this hub also carries bladder-related afferent information in a manner that would link airway and lower urinary tract control has not, to our knowledge, been established, and we do not assume it here. Over the long term, ELS has been associated with a systemic pro-inflammatory state that increases vulnerability to chronic inflammatory disease (Jessop, 2008).

### The gut–lung–bladder microbiome axis

One candidate unifying mechanism is microbiome-mediated immune programming, proposed to connect the gut to both the lung and the bladder — though, as set out below, the two links are not equally supported. Early life is the critical window for assembly of the gut microbiota and the mucosal immune system, and perturbation during this window has organ-spanning consequences.

Along the gut–lung axis, the intestinal microbiota shapes airway immunity through microbial metabolites — short-chain fatty acids and tryptophan derivatives — that modulate the Treg/Th2 balance and mucosal integrity central to allergic asthma (Zhang M. et al., 2024). This provides a mechanistic route for the same Treg-deficient, Th2-biased phenotype that ELS produces: early dysbiosis (including antibiotic-driven loss of indole-3-propionic acid–producing bacteria) durably exacerbates allergic airway inflammation (Perdijk et al., 2024). Of the three links considered here, this gut–lung relationship rests on the largest body of experimental evidence.

Along the gut–bladder axis, the bladder participates in a microbiome-centered network in which gastrointestinal, vaginal, kidney, and urinary microbiota have been proposed to communicate bidirectionally through immune, metabolic, and neural channels; dysbiosis along this “bladder-centered axis” has been associated with urinary disease (Choi et al., 2023). Whether these bladder microbial changes are causal, or instead secondary to altered voiding, local inflammation, or antibiotic exposure, has not been established, and the direction of the association remains open.

Evidence along the urinary arm carries methodological constraints that deserve explicit statement. Urine and bladder tissue are low-biomass specimens, so sequencing results are unusually sensitive to reagent and laboratory contamination, and negative controls are not uniformly reported. Collection route also matters: voided specimens sample the urethra and genital tract as well as the bladder, whereas catheterized or suprapubic specimens do not. Variation in extraction protocol, choice of 16S region, and bioinformatic pipeline limits comparison between studies, and most datasets are cross-sectional, which cannot separate a microbial cause from a microbial consequence of an already altered bladder.

The three links are therefore not equally supported. The gut–lung relationship is experimentally the strongest; the gut–bladder relationship is weaker and largely associative; and a single integrated gut–lung–bladder axis operating after early life stress has not, to our knowledge, been demonstrated in any study measuring both organs in the same animals. We therefore treat it as an emerging framework to be tested rather than an established pathway. Framed this way it yields a concrete experiment: in maternal-separation animals, characterize the gut microbiome and measure airway and lower urinary tract phenotypes in the same subjects, and ask whether a microbiome-targeted intervention — for example short-chain fatty acid restoration or defined probiotics — moves both. Because ELS has been reported to disrupt the early-life microbiome and HPA-axis–microbiome signaling, the gut remains a plausible shared upstream node, and that is what makes the prediction worth testing.

### Epigenetic reprogramming

ELS has been linked to lasting epigenetic change — DNA methylation, histone modification, and microRNA shifts — in stress- and inflammation-related genes (Nicolaides et al., 2024); altered microRNA content has also been reported in germ cells after early stress (Dickson et al., 2018). Whether these modes operate concurrently across the organ systems considered here has not been shown. Increased methylation of NR3C1 links ELS to altered autonomic regulation and respiratory sinus arrhythmia (Aghagoli et al., 2020). An analogous but distinct early-life insult makes the same point in the respiratory domain: neonatal intermittent hypoxia — a physiological, not psychosocial, stressor — drives DNA-hypermethylation-dependent oxidative-stress programming that disrupts hypoxic sensing and cardiorespiratory homeostasis into adulthood, and is preventable by a DNA-methylation inhibitor (Nanduri et al., 2012). This establishes an epigenetic route from an early physiological insult to lasting autonomic and respiratory dysfunction; whether the same route operates after maternal separation has not been shown. Germline and offspring epigenetic changes have also been reported after early stress (Lei et al., 2018), although inheritance of the multi-organ phenotype proposed here has not been demonstrated.

### A common matrix for cross-organ effects

These mechanisms share common hubs — hypothalamic and brainstem structures, the HPA axis, the amygdala, and the gut microbiome — that have been implicated in ELS effects on the respiratory and urinary systems separately (Tenorio-Lopes and Kinkead, 2021; Pervanidou and Chrousos, 2018; Bondarenko et al., 2014). A recent proposal offers one way a single early exposure could leave a durable mark in several tissues at once: that early life adversity programs tissue-resident progenitor cells, so that a common alteration is subsequently expressed by the differentiated cells of multiple organs (Mposhi and Turner, 2023). We note that this is advanced as a hypothesis rather than as an established mechanism. These candidate convergent pathways, and the peripheral-to-central influence suggested by Li-ESWT, are summarized in **Figure 3**. Individual variability means outcomes are not uniform, underscoring the need for mechanism-based stratification.

**Figure 3:**
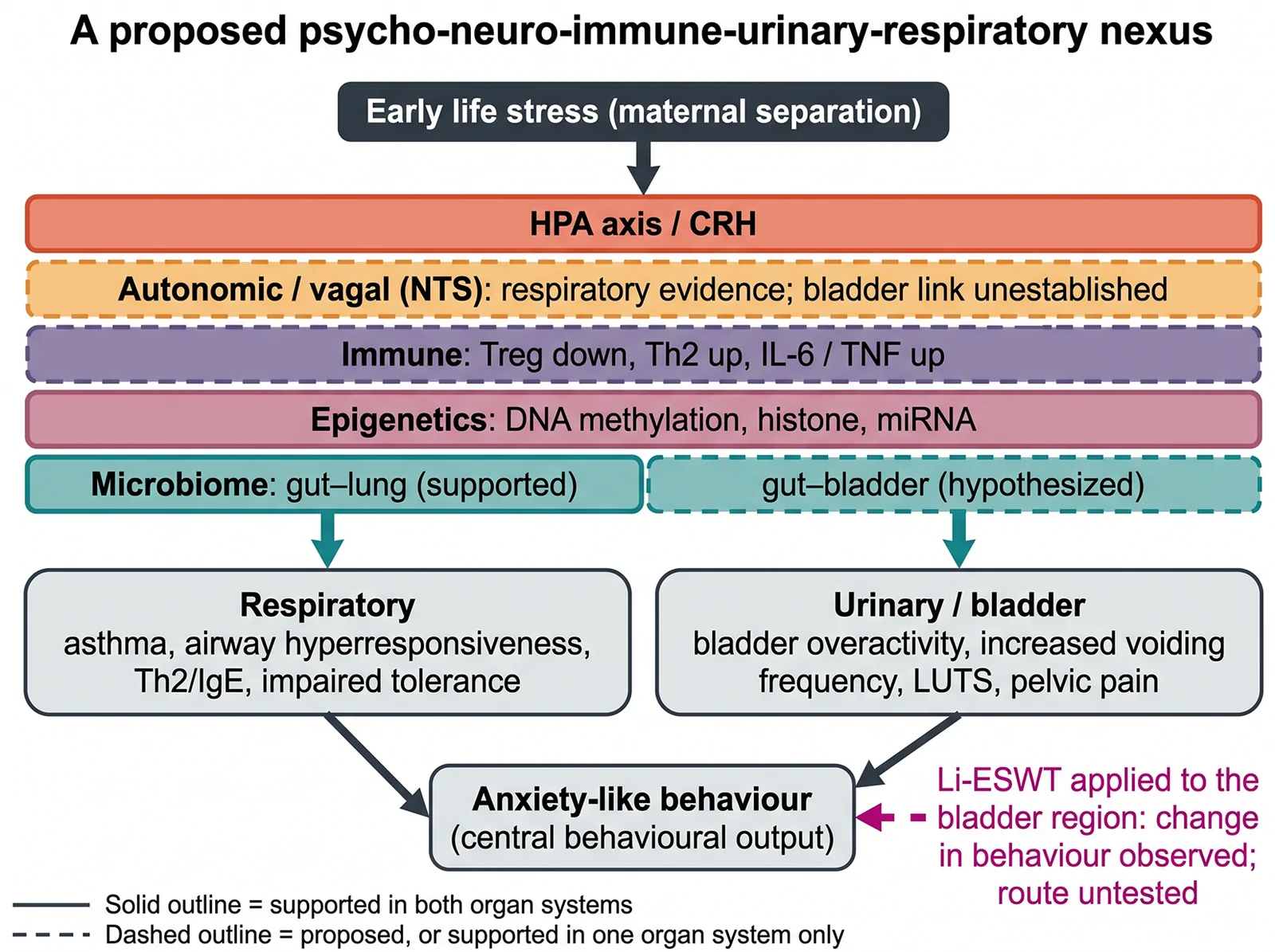
A proposed psycho-neuro-immune-urinary-respiratory nexus. Early life stress (maternal separation) may act through candidate shared mechanisms — HPA-axis/CRH signaling, autonomic and vagal (nucleus tractus solitarius) pathways, an immune shift toward reduced regulatory T cells and Th2 dominance with elevated IL-6/TNF, epigenetic reprogramming, and microbiome-mediated programming, comprising an experimentally supported gut–lung link and a hypothesized gut–bladder link — that have been associated with respiratory (airway hyperresponsiveness, Th2/IgE-biased allergic disease) or lower urinary tract (bladder overactivity, increased voiding frequency, LUTS, pelvic pain) dysfunction, or with both, together with anxiety-like behavior. In this figure a solid outline marks a mechanism supported in both organ systems and a dashed outline one that is proposed, or supported in one organ system only; this convention differs from that used in **Figure 2**, where the outline distinguishes human from rodent evidence, and the key within each figure is the one that applies to it. Not every mechanism shown is supported on both sides: in particular the vagal/nucleus tractus solitarius link to the bladder is not established, and the band is included as a candidate rather than as a demonstrated shared pathway. The dashed magenta arrow entering from the right denotes the single preclinical observation that a peripheral, bladder-directed intervention (low-intensity extracorporeal shock wave therapy, Li-ESWT) was accompanied by change in an anxiety-like behavioral outcome. This is consistent with, but does not establish, peripheral-to-central communication; the arrow is dashed because the direction, mediators, and causality remain untested.

## Li-ESWT as a candidate multi-organ intervention

### Mechanisms

Li-ESWT initiates mechanotransduction, converting mechanical stimuli into biochemical signals. In bone repair and musculoskeletal regenerative medicine, where this has been characterized, the downstream effects include proliferation, migration, and differentiation, together with angiogenesis and tissue repair (Mittermayr et al., 2021; Simplicio et al., 2020); whether the same cascade operates in the organs considered here has not been shown. It also has neuro-regenerative and immunomodulatory actions: ESWT increases neural stem-cell proliferation in injured spinal cord (Shin et al., 2018) and modulates neuroprotective microRNAs (miR-375, miR-382-5p) (Ashmwe et al., 2022); it stimulates endothelial cells via TLR3 to balance pro- (IL-6) and anti-inflammatory (IL-10) signaling (Holfeld et al., 2013; Mariotto et al., 2009).

### Lower urinary tract applications

The strongest urinary evidence is in spinal cord injury (SCI). In adult female rats after spinal cord transection, Li-ESWT to bladder and urethra (0.12 mJ/mm², 2 Hz, 300 shots ×4 = 1200) significantly improved voiding efficiency (71.2 ± 6.6% vs 51.8 ± 6.7% in controls) and raised the reduced–EUS-activity ratio during voiding (66.9 ± 5.1% vs 46.3 ± 5.1%, p = 0.017). Treatment also shortened EUS contraction duration and reduced urethral fibrosis, with an increased S-100–positive (Schwann-cell) area. Together these changes partially restored tonic EUS activity and improved detrusor-sphincter dyssynergia (Kawase et al., 2024). In an LPS-induced cystitis (interstitial cystitis/bladder pain syndrome) model, Li-ESWT (0.12 mJ/mm², 300 shots/session) improved tactile allodynia and bladder function with increased S-100–positive area (Kusakabe et al., 2024).

Most relevant to ELS, the maternal-separation experiment described above (see “Reversibility through peripheral intervention”) is the only study we are aware of that has applied bladder-directed Li-ESWT in this model. We do not repeat the bladder-weight and elevated-plus-maze results here, but the cystometric outcome deserves separate comment, because it is the finding that qualifies the picture. In the mixed-sex analysis the intercontraction interval was 4.6 ± 0.5 min in MS versus 7.7 ± 0.6 min in normal rats (F(2,44) = 6.808, p = 0.0027) — 40% shorter than control, and consistent with more frequent voiding. The treated group differed significantly from neither the normal nor the MS group. The degrees of freedom correspond to 47 animals across the three groups, and the source does not report a group mean for the treated animals in this mixed-sex analysis. Non-significance against both comparators, without a reported mean, does not establish that the interval moved toward control, and we do not claim that it did. The sex-specific analysis is more informative: in females the treated animals remained significantly below normal (4.7 ± 0.4 vs 8.0 ± 1.0 min, p < 0.05), so the voiding interval was not restored in that sex, even though the anxiety-like behavioral outcome was (Kamijo et al., 2025). To our knowledge this is the first report that Li-ESWT applied regionally to the bladder addresses organ-specific dysfunction and central behavioral alteration together in an ELS model; we treat it as a single motivating observation rather than as established efficacy.

### A theoretical extension to respiratory disease

Li-ESWT’s actions on inflammation, angiogenesis, and neural repair make respiratory application plausible but, at present, speculative. Shockwaves trigger ATP release from T cells that, via P2X7 receptors, drives p38-MAPK and FAK phosphorylation, IL-2 expression, and T-cell proliferation (Yu et al., 2010), and ESWT modulates TLR3-mediated endothelial inflammation (Holfeld et al., 2013). In principle, such immunomodulation could counter the Treg-deficient, Th2-biased airway phenotype of ELS, and neuro-regenerative effects might in principle bear on vagal respiratory control, although no cited experiment connects the two and we advance this only as a conjecture. We emphasize that no direct respiratory Li-ESWT data exist in this context; this is a hypothesis to be tested.

### Safety and parameters

It is important to distinguish two modalities that share the term “shock wave”: high-energy extracorporeal shock wave lithotripsy (ESWL, typically ~0.2–1.0 mJ/mm² or higher, used to fragment urinary stones) and low-intensity ESWT (Li-ESWT, used to stimulate tissue repair without mechanical disruption). The therapeutic effects discussed here are those of the low-intensity modality, and the two should not be conflated. We note that “low intensity” is not a single setting: the preclinical work discussed here used ~0.05–0.12 mJ/mm², whereas the clinical incontinence study cited below delivered 0.25 mJ/mm², so energies described as low-intensity in the literature span a range and are not directly comparable across studies. With that distinction noted, the favorable safety record of pediatric ESWL — where renal colic is the most common complication and long-term renal or blood-pressure sequelae are not evident — indicates that high-energy shock-wave exposure of the kidney is well tolerated in children (Schultz-Lampel et al., 1997; Zisman et al., 2022). Because the modality, energy, and target organ all differ, this does not transfer to low-intensity exposure of other organs, and it says nothing about the lung. For Li-ESWT itself, low-to-moderate energy (0.05–0.12 mJ/mm²) over multiple sessions has been reported as effective and well tolerated in the preclinical and small clinical studies discussed above; we are not aware of controlled safety data at these settings outside those contexts. Optimal parameters for stress-related dysfunction remain to be standardized, and respiratory translation in particular requires dedicated study.

### Limitations and translational barriers of Li-ESWT

Beyond the distinction between lithotripsy and low-intensity treatment, the parameters that matter are not settled. Energy flux density, pulse number, frequency, session count, and inter-session interval differ across the studies cited here, and no dose–response relationship has been established for any of the outcomes discussed. The settings used in the maternal-separation experiment (0.12 mJ/mm², 2 Hz, 200 shocks per session, nine sessions) were adopted by analogy with earlier urological protocols rather than derived from a titration. Because the wave is delivered transcutaneously to a region rather than to a defined cell population, off-target exposure of adjacent structures — bowel, reproductive organs, and neurovascular bundles — is intrinsic to the method and has not been systematically characterized in a developmental context. Effects in the ELS experiment were female-predominant, and the reason for that is unknown.

Application to the airway would face barriers that the urinary work does not. The bladder is a superficial, fluid-filled and acoustically accessible target; the lung is air-filled, and the impedance mismatch at tissue–air interfaces both limits energy delivery and raises the possibility of cavitation injury at those interfaces. Whether any setting exists that is simultaneously effective and safe for pulmonary tissue is unknown, and we are not aware of a study that has examined it.

Li-ESWT is not advanced here as the best available multi-system intervention, nor as one that has outperformed alternatives; no comparative study exists. It is the intervention we examine because it is what motivated this review, and because among the candidates it offers the most direct way to ask whether a stimulus applied at the periphery can be followed by a change in central output. As noted above, it does not by itself test the nexus proposition. Exercise acts systemically and cannot isolate a single organ. Vagal stimulation is already an intervention upon the nervous system, so a central effect would not establish that a non-neural signal can travel from a peripheral organ to the brain. Microbiome modulation acts through a humoral and immune route that Li-ESWT may not engage at all; whether bladder-directed Li-ESWT alters the microbiome has not been measured, and the two approaches should not be treated as interchangeable.

Li-ESWT, by contrast, is non-invasive, externally applied, directed toward a single peripheral target region, and non-pharmacological, which makes an observed central change harder to attribute to systemic drug exposure. It does not, however, exclude a neural route: mechanical stimulation of the bladder will activate local sensory afferents, and that is among the most obvious candidate pathways, alongside humoral, immune, endocrine, and off-target effects. Organ restriction itself has not been demonstrated. We also acknowledge a practical reason for this focus: it is the modality available to us and the one in which we have direct experimental experience. In our view the interest of the finding lies less in Li-ESWT as a therapy than in the question it raises — does a local mechanical stimulus applied to the bladder reach central behavior at all, and if so by what route? Answering that question, rather than promoting the modality, is what we take to be the useful next step.

## Future directions

### Models for simultaneous multi-organ evaluation

The field needs ELS models that evaluate respiratory and urinary function in the same animal. Enabling technologies include validated rodent urodynamics (Schneider et al., 2017), simultaneous bladder-pressure/neural recording in freely moving mice (Yao et al., 2019), high-precision implantable bladder-pressure microsystems (Li et al., 2020), and multi-organ-on-chip platforms (Zhao et al., 2019). Model choice also matters: rats and mice differ in electrophysiological yield, genome-editing toolkits, xenobiotic-metabolizing networks, and inflammatory responses (Ellenbroek and Youn, 2016; Karthikeyan et al., 2016; Kidd et al., 2001).

### Integrated interventions and biomarkers

In a single-arm, open-label clinical study of 50 women with stress urinary incontinence, Li-ESWT (0.25 mJ/mm², 3000 pulses, once weekly for 8 weeks) reduced urine leakage and improved questionnaire-based quality of life (Long et al., 2020). Separately, in preclinical work, Li-ESWT has been reported to increase neural stem-cell proliferation in injured spinal cord (Shin et al., 2018) and to alter circulating neuroprotective microRNAs in the same setting (Ashmwe et al., 2022); whether those changes mediate functional recovery was not established. Combination strategies are promising: aerobic exercise raises BDNF and offsets ELS effects on neuroplasticity (Campbell et al., 2022), and environmental enrichment restores hippocampal neurogenesis impaired by early stress (Rule et al., 2020). Multi-system assessment should pair central and autonomic readouts (heart rate variability, cortisol/catecholamines, IL-6/CRP; Shah et al., 2024) with airway-relevant urinary proteins measurable by multiplex mass spectrometry (e.g. CC16, osteopontin; Nauwelaerts et al., 2021). Airway and endothelial injury can also be indexed from serum: VEGF-A tracks COVID-19 severity and correlates with surfactant protein-D and neutrophil counts (Tsuji et al., 2024). Readouts of this kind are accessible enough to be added to a multi-organ stress panel, although they have not been examined in an ELS context.

### Translational validity

Animal models permit controlled study of childhood stress and reveal therapeutic targets (Battaglia and Khan, 2018). Bridging designs that pair mechanistic animal work with human cohorts — and that draw on work showing epigenetic marks to be pharmacologically modifiable in other disease contexts (Patnaik et al., 2023) — are needed to move the nexus framework from hypothesis toward the clinic.

## Limitations

Most of the mechanistic evidence reviewed here comes from rodent maternal-separation models. These models offer experimental control that human studies cannot, but the mapping from a defined separation protocol to the heterogeneous adversities captured by adverse childhood experience instruments is loose: separation is a single, timed, reproducible exposure, whereas human adversity is chronic, multi-dimensional, and typically correlated with the circumstances that follow it. Findings obtained in rodents should therefore be read as generating hypotheses about human disease rather than as establishing it, and this review does not claim otherwise.

On the human side, the exposure itself is measured inconsistently. Adverse childhood experience instruments differ in the events they count, the age windows they cover, and their reliance on retrospective recall, so effect sizes are not directly comparable between cohorts. The associations reported here are also open to confounding that the source studies could not fully remove: socioeconomic position, maternal mental health, and environmental exposures such as air pollution and tobacco smoke each independently affect airway and lower urinary tract outcomes, and each tends to co-occur with early adversity. Where mediation has been estimated — for example the roughly half of the trauma–incontinence association attributable to mental-health pathways in LURN — the remainder is unexplained.

Sex is a systematic source of variability rather than a nuisance. In the urinary literature females are generally the more affected sex, whereas in the respiratory literature males show the larger ventilatory phenotype, and the epidemiological pattern differs again according to whether the exposure is prenatal or postnatal. A framework that treats the two organ systems as expressions of one upstream state has to accommodate this divergence, and at present it does not explain it; glucocorticoid signaling is itself sexually dimorphic (Martinez et al., 2024), which may be part of the answer but has not been tested in this context. Human biomarker validation is a further gap: the candidate readouts discussed here — cortisol and catecholamine measures, heart rate variability, IL-6 and CRP, and urinary proteomic panels — have not been shown to track ELS-related pathology across both organ systems in the same cohort. A further constraint applies specifically to the epigenetic readouts. Human studies of childhood adversity have relied largely on blood and saliva, and methylation patterns are cell-type specific, so markers obtained from peripheral tissues may not represent the tissue of interest. On this basis the human evidence for epigenetic programming after adversity has been judged only partially supportive (Krause et al., 2020).

Finally, the therapeutic claims in this review are its most provisional element. The evidence that a peripheral intervention is accompanied by change in a central behavioral endpoint rests on a single preclinical study, and the extension to the airway is a reasoned inference rather than an observation. No study we are aware of has measured respiratory and lower urinary tract phenotypes in the same ELS-exposed animals, which is the minimum design required to test the central proposition of this review. Until that is done, the nexus remains a hypothesis and the multi-organ therapeutic strategies it suggests remain untested.

## Conclusion

ELS produces dysfunction in more than one organ system, and that pattern may not be fully captured by studying organs separately. In maternal separation models, ELS impairs respiratory tolerance (regulatory T-cell suppression, Th2 bias; Ouchi et al., 2018) and, in parallel, alters lower urinary tract function and behavior (Kamijo and Miyazato, 2023), plausibly through shared HPA-axis, autonomic, microbiome (gut–lung–bladder), and epigenetic mechanisms. The observation that a peripheral intervention — bladder-directed Li-ESWT — improved organ dysfunction and central behavior together (Kamijo et al., 2025) is, to date, single-study preclinical evidence; we treat it as an observation that motivates investigation of possible peripheral-to-central communication, not as a demonstration of it and not as validation of a multi-organ therapy. Its wider interest lies in the possibility it raises: that other organs which may share elements of the same early-life programming — the airway among them — might also be reachable from the periphery. Testing that possibility will require integrated multi-organ models, shared biomarker panels, and human studies designed to ask whether the associations reviewed here are causal.
